# Supplementary material for: Analysis of the Gut Microbiome and Dietary Habits in Metastatic Melanoma Patients with a Complete and Sustained Response to Immunotherapy
Source: Cancers (Basel). 2023 Jun 4;15(11):3052. doi: 10.3390/cancers15113052 (PMC10252899; doi:10.3390/cancers15113052)
Supplement: Supplementary file 1 [file cancers-15-03052-s001.zip › cancers-2401678-supplementary.pdf]

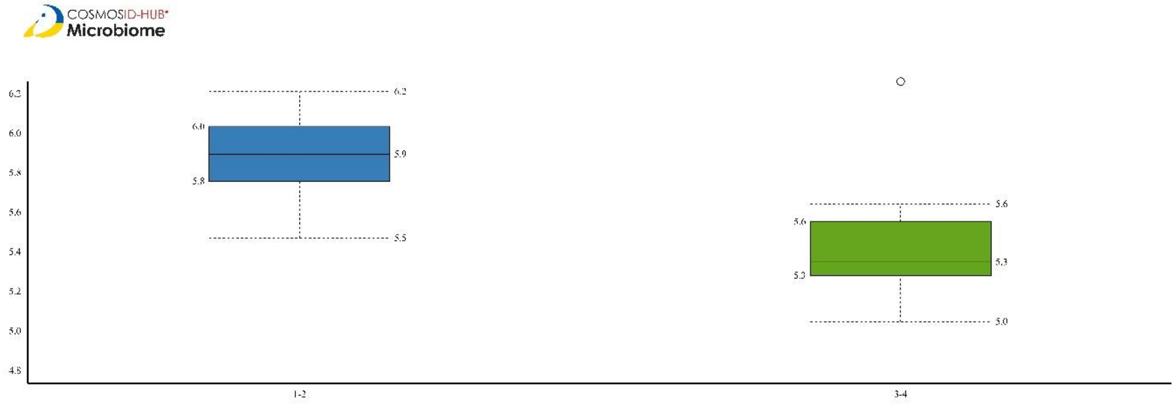

Supplementary Figure S1: A box plot showing the difference in alpha-diversity (Shannon) between metastatic melanoma patients with sustained complete response on immunotherapy, based on the initial number of metastatic sites ($p<0.05$). Green = patients with 3+ metastatic sites, blue = patients with 1-2 metastatic sites.

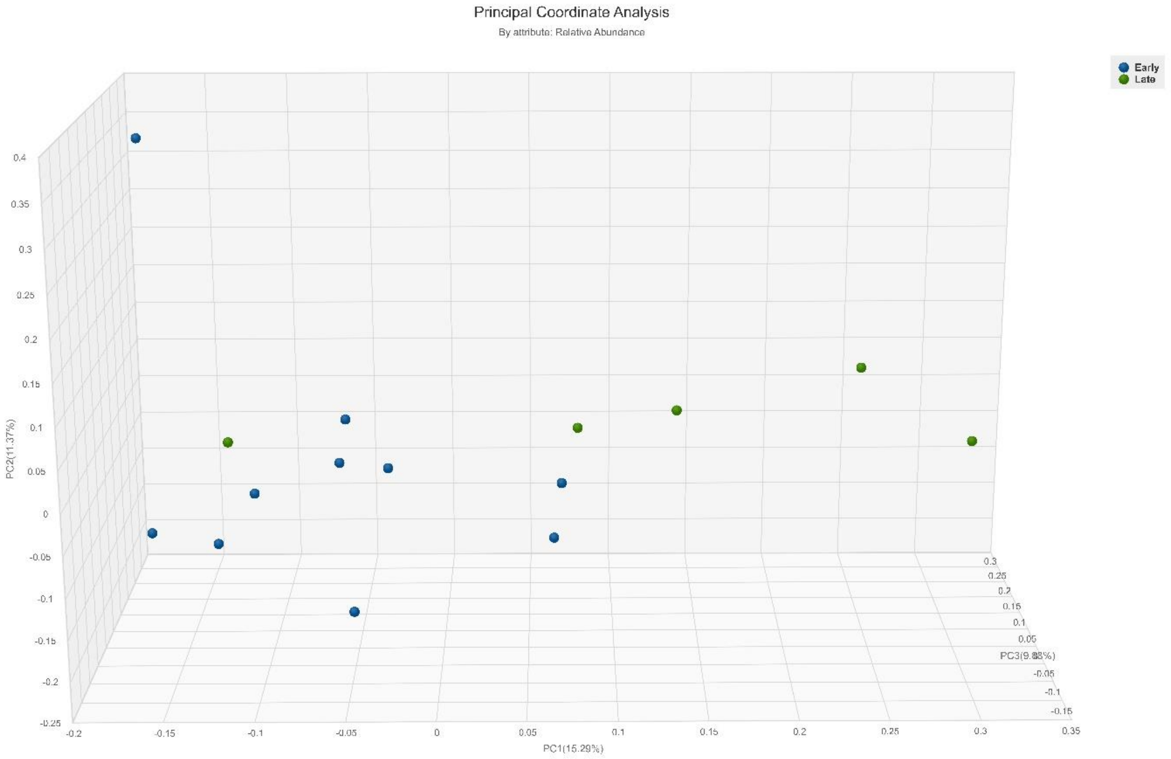

Supplementary Figure S2: A principal coordinate analysis graph (Jaccard) showing the difference in beta-diversity between metastatic melanoma patients who required more or less than 9 months to achieve complete response on immunotherapy ($p=0.02$). Green = late responders, blue = early responders.

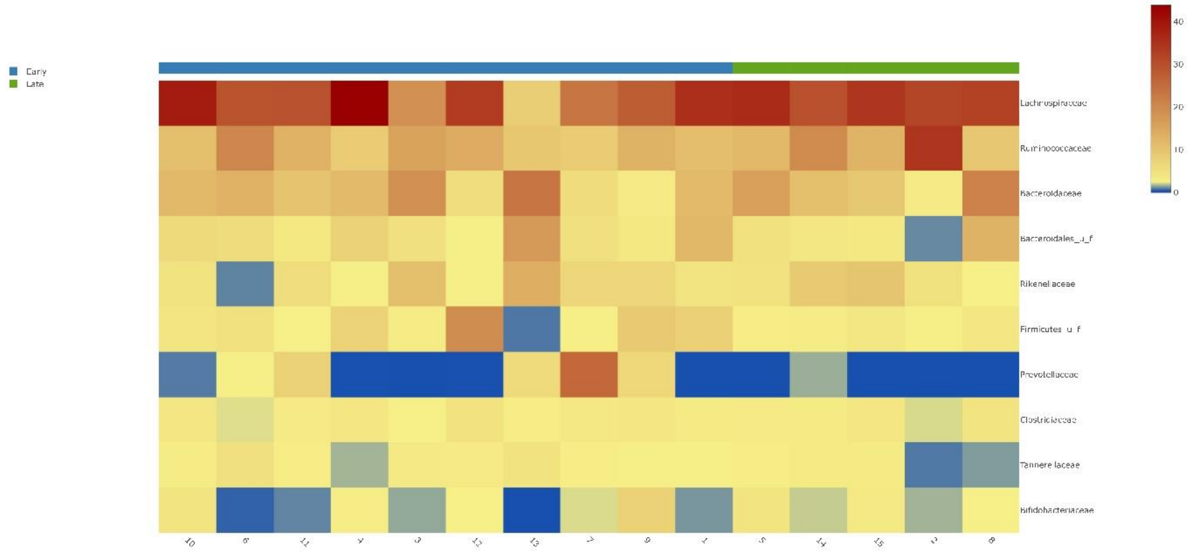

Supplement Figure S3: A heatmap view of the difference in abundance score of the phylum level for all patients, divided to early and late responders, based on whether the complete response to immunotherapy occurred before or after 9 months since the start of immunotherapy. Green = late responders, blue = early responders.

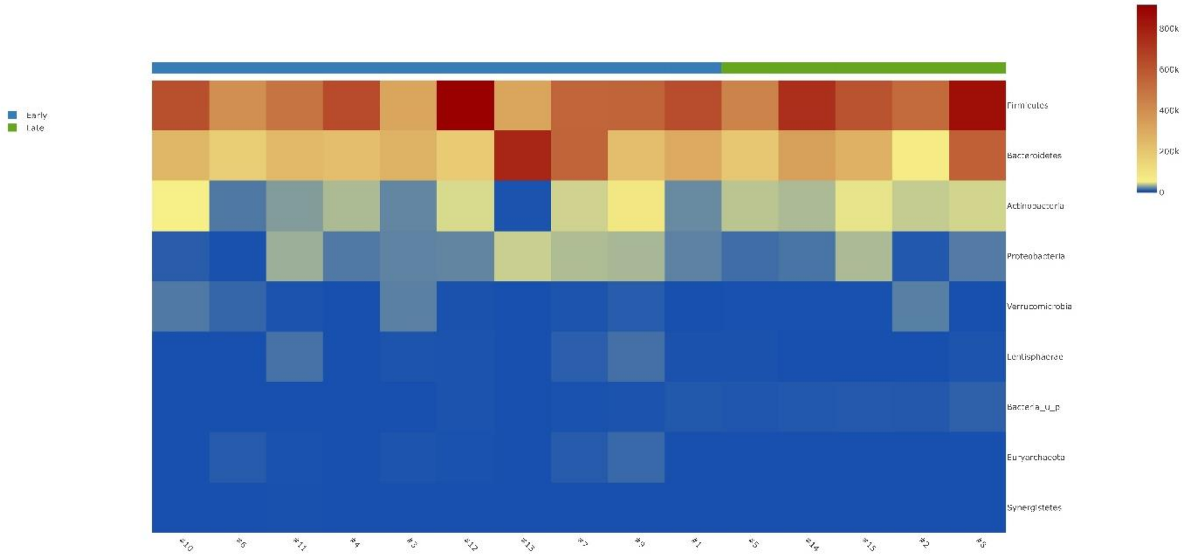

Supplement Figure S4: A stacked bar view of the difference in relative abundance of the phylum level for all patients, divided to early and late responders, based on whether the complete response to immunotherapy occurred before or after 9 months since the start of immunotherapy.

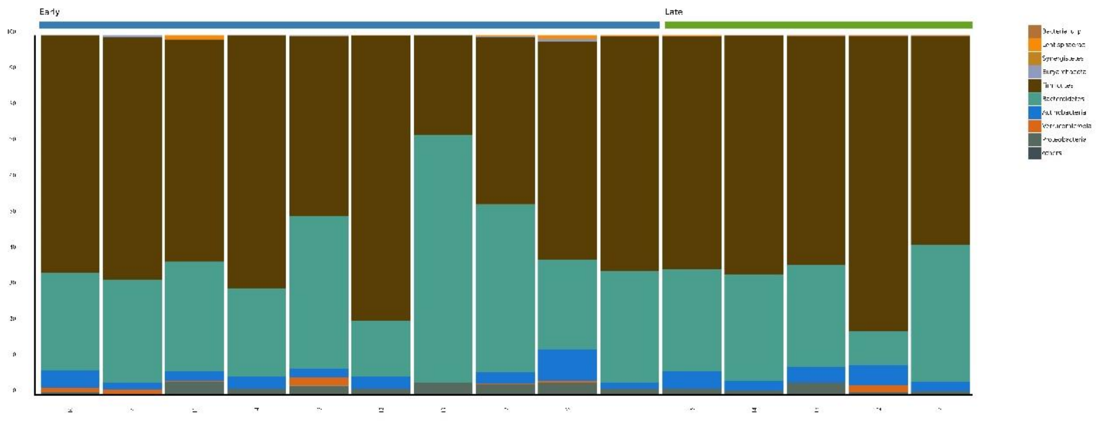

Supplement Figure S5: A heatmap view of the difference in relative abundance of the ten most common bacterial families for all patient cohort, divided to early and late responders, based on whether the complete response to immunotherapy occurred before or after 9 months since the start of immunotherapy. Green = late responders, blue = early responders.

Supplementary Table S1: Differences between metastatic melanoma patients with sustained complete response to immunotherapy depending on the time-to-response (demographics and habits)

Parameter	Late responders	Early responders	p-value
Gender			
Female (%)	1 (33.3)	2 (66.6)	0.4936
Male (%)	4 (33.3)	8 (66.6)	
Marital status			
Married (%)	3 (30)	7 (70)	0.846
Not married (%)	2 (40)	3 (60)	
Weekly hours spend socializing			
1 (%)	1 (50)	1 (50)	0.8740
2-5 (%)	1 (50)	1 (50)	
5-10 (%)	2 (28.6)	5 (71.4)	
11-20 (%)	1 (25.0)	3 (75.0)	
Highest education level			
Elementary school (%)	1 (100)	0 (0)	0.2451
High school (%)	3 (37.5)	5 (62.5)	
Higher education (%)	1 (16.7)	5 (83.3)	
Professional status			
Retired (%)	5 (41.7)	7 (58.3)	0.4936
Working (%)	0 (0)	3 (100)	
Breastfed			
No (%)	1 (50)	1 (50)	0.7327
Yes (%)	4 (33.3)	8 (66.7)	
Smoking status			
Non-smoker (%)	4 (40)	6 (60)	0.8465
Smoker (%)	1 (20)	4 (80)	
Prescription drug use			
No (%)	0 (0)	2 (100)	0.7883
Yes (%)	5 (38.5)	8 (61.5)	
Blood pressure drug use			
No (%)	2 (33.3)	4 (66.7)	0.5762
Yes (%)	3 (38.5)	6 (61.5)	
Antidiabetic drugs			

No (%)	4 (33.3)	8 (66.7)	0.4936
Yes (%)	1 (33.3)	2 (66.7)	
Thyroid drug use			
No (%)	4 (30.8)	9 (69.2)	0.7883
Yes (%)	1 (50)	1 (50)	
Corticosteroid use			
No (%)	4 (30.8)	9 (69.2)	0.7883
Yes (%)	1 (50)	1 (50)	
Hypolipemic use			
No (%)	5 (41.7)	7 (58.3)	0.4936
Yes (%)	0 (0)	3 (100)	
Previous use of supplements			
No (%)	3 (30)	7 (70)	0.8465
Yes (%)	2 (40)	3 (60)	
Average daily sleep			
>9 hours (%)	0 (0)	1 (100)	0.4966
7-8 hours (%)	4 (44.4)	5 (55.6)	
4-6 hours (%)	1 (20)	4 (80)	
The cut-off for late responders was 9 or more months to reach complete response. Bolded values denote statistical significance (p<0.05).			

Supplementary Table S2: Differences between metastatic melanoma patients with sustained complete response to immunotherapy depending on the time-to-response (disease, treatment, and response)			
Parameter	Late responders	Early responders	p-value
BRAF mutation status			
Negative	4 (40%)	6 (60%)	0.8465
Positive	1 (20%)	4 (80%)	
Initial melanoma T stage			
2a	0 (0%)	1 (100%)	0.3787
2b	0 (0%)	1 (100%)	
3a	0 (0%)	3 (100%)	
3b	0 (0%)	2 (100%)	
4a	2 (66.7%)	1 (50%)	
4b	2 (66.7%)	1 (50%)	
Unknown	1 (50%)	1 (50%)	
Initial melanoma N stage			
0	1 (20%)	4 (80%)	0.6769
1a	1 (33.3%)	2 (60.7%)	
1c	1 (100%)	0 (0%)	
1 – unspecified	1 (33.3%)	2 (66.7%)	
2	0 (0%)	1 (100%)	
Unknown	1 (50%)	1 (50%)	
Type of immunotherapy received			
Dual	1 (33.3%)	2 (66.7%)	0.4936
Mono	4 (33.3%)	8 (66.7%)	
Line of immunotherapy			
1	5 (35.7%)	9 (64.3%)	0.7144
2	0 (0%)	1 (100%)	
Initial pseudoprogression			
No	4 (30.8%)	9 (69.2%)	0.7883
Yes	1 (50%)	1 (50%)	
Level of biomarkers			
Initial LDH	215.75 (96.94)	236.00 (75.03)	0.6875
Final LDH	170.6 (30.75)	197.50 (13.28)	0.2287
Initial S100	0.167 (0.11)	0.34 (0.78)	0.7341
Final S100	0.033 (0.01)	0.040 (0.023)	0.3913
Time periods			
Time to M1 disease	1.32 (0.89)	1.58 (1.02)	0.6375
Time to any response	4.82 (3.25)	3.70 (1.17)	0.6242
**Time to complete response**	**13.04 (2.97)**	**4.90 (1.93)**	**<0.0001**
The cut-off for late responders was 9 or more months to reach complete response. Bolded values denote statistical significance (p<0.05).			

Supplementary Table S3. The difference in alpha and beta diversity of fecal microbiome for different patient characteristics.				
	Alpha diversity		Beta diversity	
Patient characteristic	Chao1 (p)	Simpson (p)	Jaccard (p)	Bray Curtis (p)
Age[1]	0.12 (0.91)	-1.06 (0.29)	1.14 (0.17)	0.91 (0.62)
Gender (m/f)	0.29 (0.77)	-1.15 (0.25)	0.91 (0.64)	0.69 (0.85)
Average daily activity[1]	-0.23 (0.82)	-1.85 (0.06)	1.08 (0.27)	0.93 (0.65)
Breastfed status (y/n)	0.14 (0.89)	-0.29 (0.77)	0.88 (0.75)	0.60 (0.94)
Sleep[2]	-0.49 (0.62)	-0.49 (0.62)	0.87 (0.80)	0.64 (0.94)
BRAF status[3]	0.00 (1.00)	0.73 (0.46)	1.05 (0.32)	1.37 (0.10)
Type of immunotherapy[4]	1.01 (0.31)	0.00 (1.00)	0.92 (0.63)	0.80 (0.73)

Number of metastatic sites[5]	**1.96 (0.05)**	**2.08 (0.04)**	1.18 (0.13)	0.74 (0.87)
Time to metastasis[1]	0.23 (0.82)	0.46 (0.64)	1.01 (0.48)	0.65 (0.93)
Bone metastases (y/n)	-0.36 (0.71)	0.12 (0.90)	1.25 (0.07)	0.94 (0.55)
Cutaneous / subcutan. metastases (y/n)	-0.82 (0.41)	0.59 (0.56)	1.06 (0.34)	0.89 (0.64)
Lung metastases (y/n)	-0.59 (0.56)	-0.59 (0.56)	1.01 (0.47)	1.31 (0.11)
Lymph node metastases (y/n)	0.58 (0.56)	-0.34 (0.73)	1.13 (0.17)	1.06 (0.39)
Recent use of analgetics (y/n)	0.39 (0.69)	0.52 (0.60)	0.89 (0.76)	1.09 (0.33)
Initial nodal disease (y/n)	0.07 (0.95)	0.20 (0.84)	1.02 (0.41)	1.07 (0.33)
Initial LDH[1]	0.43 (0.67)	-0.14 (0.89)	1.03 (0.38)	0.80 (0.77)
Initial S100[1]	1.22 (0.22)	1.54 (0.12)	1.19 (0.14)	1.24 (0.19)
Time to complete response (CR)[6]	0.24 (0.81)	0.49 (0.62)	**1.43 (0.02)**	**1.65 (0.02)**
Time to partial response (PR)[7]	-0.51 (0.61)	-0.17 (0.86)	0.80 (0.89)	0.84 (0.57)
BP drug use (y/n)	-0.47 (0.64)	-0.47 (0.64)	0.88 (0.78)	1.07 (0.35)
Antidiabetic drug use (y/n)	0.87 (0.38)	1.29 (0.19)	1.20 (0.16)	1.08 (0.35)
Hypolipemic drug use (y/n)	0.00 (1.00)	0.29 (0.77)	1.19 (0.15)	1.39 (0.15)
Supplement use (y/n)	-0.52 (0.60)	0.26 (0.79)	0.91 (0.68)	0.87 (0.62)
Use of antibiotics with immunotherapy (y/n)	1.43 (0.15)	1.83 (0.07)	1.24 (0.09)	0.95 (0.49)
Obesity[8]	-1.59 (0.11)	-0.86 (0.39)	1.09 (0.26)	1.27 (0.15)
DII[1]	0.78 (0.43)	1.43 (0.15)	**1.43 (0.01)**	1.38 (0.14)
Industrial food use (y/n)	-0.94 (0.35)	-0.82 (0.41)	1.00 (0.47)	0.51 (0.99)
Number of daily meals (</> 4)	0.52 (0.60)	1.04 (0.29)	0.89 (0.72)	0.62 (0.95)
Salt[1]	-1.50 (0.13)	0.00 (1.00)	0.87 (0.77)	0.92 (0.59)
High fibre diet (</> 20g/day)	-0.58 (0.56)	1.01 (0.31)	0.75 (0.94)	0.47 (0.99)
Use of HG milk (y/n)	-0.91 (0.36)	-0.78 (0.43)	1.14 (0.19)	1.29 (0.18)
Use of HG fruit (y/n)	-0.35 (0.73)	-0.92 (0.35)	1.07 (0.29)	1.00 (0.45)
Use of HG vegetable (y/n)	0.26 (0.79)	0.52 (0.60)	0.78 (0.93)	0.56 (0.97)
Use of HG meat (y/n)	-0.25 (0.81)	0.85 (0.39)	0.79 (0.91)	0.89 (0.61)
Breakfast time (early *vs.* late)	0.14 (0.89)	0.00 (1.00)	0.88 (0.76)	1.06 (0.37)
Dinner time (early *vs.* late)	-1.16 (0.24)	-1.68 (0.09)	0.75 (0.95)	0.61 (0.96)

Bolded values denote statistical significance (p<0.05).
[1] Based on the median value. [2] More or less than 7 hours average. [3] Mutated *vs.* wild type. [4] Mono *vs.* dual. [5] Up to two *vs.* three and more different organs. [6] Based on the difference early vs. late complete response to immunotherapy with the cut-off of 9 months. [7] Based of whether partial response was present on initial scan. [8] Based on BMI more or less than 25.
CR = complete response. PR = partial response. DII = Dietary inflammatory index. HG = home-grown. M/f = male *vs.* female. Y/n = yes vs. no.

Supplementary Table S4: The difference in relative abundance of specific bacterial families, previously associated with a significant effect on immunotherapy in melanoma patients, between early and late metastatic melanoma responders to immunotherapy

	Early responders (N=10)[a]		Late responders (N=5)[a]		
	Median	25 - 75 Percentile	Median	25 - 75 Percentile	p-value
Akkermansiaceae[19]	0.0950	0.000 - 1.060	0.0200	0.0150 - 0.550	0.49
Bacteroidaceae[19]	11.540	5.600 - 13.000	10.770	7.610 - 17.413	1.00
Bifidobacteriaceae[9,17]	1.565	0.730 - 2.740	2.200	1.608 - 3.540	0.39
Clostridiales[18]	1.900	1.680 - 2.800	2.820	1.683 - 3.285	0.54
Coriobacteriaceae[17]	0.445	0.320 - 0.810	0.180	0.165 - 0.520	0.33
Lachnospiraceae[16,17]	29.425	23.430 - 35.610	32.410	31.160 - 35.000	0.33
Lactobacillaceae [19]	0.755	0.590 - 1.040	0.830	0.180 - 1.920	1.00
Prevotellaceae[11,19]	**1.555**	**0.0100 - 6.390**	**0.000**	**0.000 - 0.323**	**0.046**
Ruminococcceae [4,11,17]	11.740	9.100 - 13.900	12.590	11.180 - 23.330	0.39

[a] The cut-off for late responders was 9 or more months to reach complete response. Bolded values denote statistical significance (p<0.05).

Supplementary Table S5: The difference in abundance score of specific bacterial phyla previously associated with a significant effect on immunotherapy in melanoma patients between early and late metastatic melanoma responders to immunotherapy

	Early responders (N=10)[a]		Late responders (N=5)[a]		
	Median	25 - 75 Percentile	Median	25 - 75 Percentile	p-value
Actinobacteria[10]	26237.460	15556.300 - 39405.390	35281.210	32687.532 - 39175.553	0.39
Bacteroidetes[10]	250801.885	225039.200 - 299399.010	271869.900	160070.385 - 388830.420	0.90
Firmicutes[10,16]	548194.620	397259.880 - 634760.690	608623.350	504873.498 - 773533.362	0.46
Proteobacteria[10]	15001.600	11779.770 - 29677.620	10153.390	6799.110 - 16804.502	0.27

[a] The cut-off for late responders was 9 or more months to reach complete response.

Supplementary Table S6: Characteristics of the metastatic melanoma patients with a sustained complete response to immunotherapy confirmed on PET/CT (dietary habits and exercise)

Patient	Weight (kg)	Height (m)	BMI	BMI class	DII	MDS	Mifflin St Jeor equation (kCal)	Energy intake (kCal)	Number of daily meals	Regular use of HG food	HG milk	HG fruit	HG vegetable	HG meat	Regular use of industrial pre-made food	Use of artificial sweet-eners	Time of breakfast (hour of the day)	Time of lunch (hour of the day)	Time of dinner (hour of the day)
#1	83	1.72	28.06	OW	3.10	4.50	1910.00	2207.99	3	yes	no	no	yes	yes	Some-times	no	6 to 7	14 to 15	18-19
#2	120	1.87	34.32	obese	-0.49	5.25	2373.75	1999.03	4	yes	yes	yes	yes	yes	no	no	9	12	17
#3	85	1.8	26.23	OW	2.79	5.50	1980.00	1992.36	3	yes	no	yes	yes	yes	no	no	7 to 8	16	no dinner
#4	79	1.86	22.84	normal	-1.82	9.25	1957.50	2473.61	5	yes	no	yes	yes	yes	no	no	6 to 7	12	19-20
#5	60	1.61	23.15	normal	3.79	5.50	1445.25	1708.83	6	no	no	no	no	no	no	no	7 to 8	12:30	18:30-20
#6	90	1.72	30.42	obese	1.52	6.50	1814.00	1657.03	5	yes	yes	yes	yes	yes	no	no	8	13	18
#7	78	1.76	25.18	OW	3.10	5.75	1885.00	2142.17	4	yes	no	no	yes	no	no	no	7 to 8	12	15-16
#8	85	1.82	25.66	OW	-0.17	8	1992.50	1944.77	2	yes	no	no	yes	yes	Some-times	no	no breakfast	12	18
#9	75	1.69	26.26	OW	1.64	5.25	1645.25	2108.98	4	yes	yes	yes	yes	yes	no	no	6 to 7	12:30	20
#10	73	1.73	24.39	normal	1.18	5.25	1816.25	1972.72	4	yes	no	yes	yes	yes	no	no	7	11	17
#11	80	1.74	26.42	OW	2.37	4.75	1892.50	1818.13	4	yes	no	yes	no	yes	Some-times	no	7 to 8	11:30-13	19
#12	98	1.76	31.64	obese	3.44	4.25	2085.00	1804.97	5	yes	no	no	no	no	regularly	yes	9	14	18
#13	76	1.76	24.54	normal	2.51	5.50	1865.00	2188.21	4	yes	no	yes	yes	yes	no	no	7 to 8	12	18:30
#14	117	1.91	32.07	obese	1.68	6.25	2368.75	2204.94	3	yes	yes	no	yes	no	Some-times	no	9	14:30	19:30
#15	74	1.75	24.16	normal	-1.35	7	1838.75	2446.00	5	no	no	no	no	no	Some-times	no	7 to 8	12	19

DII = dietary inflammatory index. HG = home-grown. OW = overweight. MDS = Mediterranean Diet Score

Supplementary Table S7: The difference in food intake between metastatic melanoma patients with early or late response to immunotherapy.

	Cohort				Between-Subjects Effects	Within-Subjects Effects
	Early responders (N=10)[a]		Late responders (N=5)[a]			
	Mean	SD	Mean	SD		
Alcohol (% caloric intake)	1,647	4,2101	3,500	4,9029	0,379	0,406*
Alcohol (g)	4,149	10,3716	10,992	14,5049	0,237	0,660*
Alcoholic drinks (g)	25,536	63,6602	118,572	190,4176	0,090	0,517*
Anthocyanidin (mg)	131,328	209,4609	266,063	333,6942	0,094	0,340**
Beta-carotene (mg)	3234,557	3519,4818	4577,746	4871,5676	0,396	0,079*
Plant fibre (% recommended daily intake)	102,170	43,7425	90,161	31,2327	0,540	0,914*
Plant fibre (g)	25,543	10,9359	22,541	7,8080	0,540	0,914*
Ca (% recommended daily intake)	105,642	45,3124	94,750	38,1569	0,555	0,658*
Ca (mg)	1056,418	453,1296	947,504	381,5836	0,555	0,658*
Cinnamon (g)	0,0500	0,2481	0,000	0,0000	0,380	0,448**
Curry (g)	0,000	0,0000	0,000	0,0000	-	-
Garlic (g)	3,482	2,3519	5,129	7,8660	0,320	0,329**
DII	1,981	1,9205	0,693	2,3553	0,192	0,605*

Added fats (g)	17,937	12,1490	24,053	13,2629	0,231	0,563*
Ginger (g)	0,00900	0,05692	0,000	0,0000	0,500	0,365**
Energy intake (kcal)	2036,617	289,4360	2060,717	438,0011	0,864	0,654*
Energy drinks (ml)	0,000	0,0000	0,000	0,0000	-	-
Energy (% recommended daily intake)	93,210	14,8473	93,617	15,8333	0,949	0,513*
Energy needs (BMR i TA)	2639,070	156,9961	2747,510	600,5819	0,618	-
Fe (% recommended daily intake)	191,486	167,2116	180,213	59,7302	0,838	0,247*
Fe (mg)	21,064	18,3935	19,823	6,5700	0,838	0,247*
Flavan-3-ol (mg)	19,357	19,0778	14,491	13,2824	0,488	0,768**
Flavones (mg)	3,616	3,9908	9,073	9,1212	0,027	0,640*
Flavonols (mg)	109,447	91,4019	80,232	50,1669	0,247	0,773*
Flavonones (mg)	14,582	33,9864	14,180	35,4782	0,976	0,289**
Folate (% recommended daily intake)	85,540	48,7608	96,431	51,4771	0,621	0,895*
Folate (mg)	273,725	156,0367	308,575	164,7256	0,621	0,895*
Carbonates drinks (g)	0,000	0,0000	0,000	0,0000	-	-
Legumes (% recommended daily intake)	87,741	152,4824	68,333	168,4024	0,737	0,993*
Legumes (g)	39,483	68,6166	30,750	75,7815	0,737	0,993*
I (% recommended daily intake)	38,150	19,2814	48,137	44,9610	0,491	0,913**
I (mcg)	57,225	28,9219	72,207	67,4431	0,491	0,913**
Eggs (% recommended daily intake)	230,303	373,7357	230,770	439,4162	0,996	0,918*
Eggs (g)	29,939	48,5856	30,000	57,1241	0,996	0,918*
K (% recommended daily intake)	96,722	26,9172	107,638	27,1342	0,398	0,815*
K (mg)	3385,267	942,1071	3767,328	949,7048	0,398	0,815*
Coffee (g)	85,000	72,6777	95,000	91,6228	0,810	0,244*
Cumin (g)	0,000	0,0000	0,000	0,0000	-	-
Cloves (g)	0,000	0,0000	0,000	0,0000	-	-
Caffeine (mg)	181,574	154,2206	202,086	194,3265	0,816	0,248*
Cholesterol (% recommended daily intake)	119,291	67,1259	125,563	80,4426	0,768	0,743*
Cholesterol (mg)	357,873	201,3762	376,688	241,3305	0,768	0,743*
Potato (g)	103,036	136,8100	188,286	142,2395	0,067	0,540*
Turmeric (mg)	0,000	0,0000	0,000	0,0000	-	-
Onion (g)	6,450	20,6086	1,300	5,8138	0,445	0,661**
Thyme (g)	0,0750	0,1256	0,0300	0,07327	0,364	0,741*
MDS	5,650	1,8053	6,400	2,0622	0,324	0,820*
Meat (% recommended daily intake)	207,178	142,2026	210,137	193,0323	0,955	0,947*
Meat (g)	176,101	120,8717	178,616	164,0778	0,955	0,947*
Mg (% recommended daily intake)	107,675	43,9047	105,151	27,4672	0,895	0,682*
Mg (mg)	376,861	153,6661	368,030	96,1360	0,895	0,682*
Dairy products (% recommended daily intake)	162,999	104,0221	90,343	92,8772	0,150	0,614*
Dairy products (g)	407,500	260,0545	225,857	232,1931	0,150	0,614*
Monounsaturated fatty acids (% caloric intake)	14,383	4,0007	14,209	4,9839	0,895	0,659*
Monounsaturated fatty acids (g)	32,887	10,5157	32,681	14,3541	0,960	0,902*
Na (% recommended daily intake)	182,925	62,4301	193,722	53,2671	0,656	0,705**
Na (mg)	3658,479	1248,6008	3874,423	1065,3170	0,656	0,705**
Niacin (% recommended daily intake)	122,270	52,3610	140,950	57,1036	0,378	0,286*
Niacin (mg)	11,389	4,6682	13,306	5,6780	0,328	0,337*
Omega-3 (g)	0,544	0,5260	0,907	1,1331	0,331	0,526**
Omega-6 (g)	0,691	0,5219	0,673	0,5862	0,911	0,155*
Nuts (% recommended daily intake)	29,762	61,6380	17,500	43,7547	0,578	0,697*
Nuts (g)	8,929	18,4915	5,250	13,1264	0,578	0,697*
Oregano (g)	0,0975	0,1804	0,0300	0,07327	0,205	0,902**
Other polyphenols (mg)	330,263	202,4377	362,603	166,5507	0,704	0,327*
P (% recommended daily intake)	163,126	42,1422	146,394	37,3145	0,382	0,264*
P (mg)	1631,256	421,4211	1463,944	373,1492	0,382	0,264*
Pepper (mg)	0,442	0,3637	0,305	0,3187	0,341	0,622*
Polyunsaturated fatty acids (% caloric intake)	5,818	2,4680	8,386	4,2103	0,099	0,541*
Polyunsaturated fatty acids (g)	12,968	5,0158	19,189	10,9417	0,105	0,540*

Vegetables without potato (g)	272,670	174,7016	372,036	214,5530	0,116	0,833*
Proteins (% caloric intake)	19,536	2,6378	18,494	3,3775	0,273	0,692*
Proteins (% recommended daily intake g/kg BM)	178,100	50,5530	133,946	23,1728	0,005	0,365**
Proteins (g)	91,658	24,7117	92,703	31,4135	0,926	0,818**
Retinol (vit. A (% recommended daily intake))	104,519	196,3248	75,189	44,9043	0,502	0,306**
Retinol (vit. A (mcg))	700,276	1763,3724	428,583	255,9531	0,492	0,322**
Fish (% recommended daily intake)	44,643	199,4543	216,735	469,6637	0,127	0,814**
Fish (g)	15,625	69,8091	75,857	164,3817	0,127	0,814**
Rosemary (g)	0,0175	0,1107	0,0600	0,1875	0,258	0,923*
Se (% recommended daily intake)	42,216	29,2344	69,326	71,3885	0,173	0,138*
Se (mg)	29,553	20,4640	48,530	49,9727	0,173	0,138*
Sweets (g)	54,170	56,9123	14,590	23,4487	0,040	0,664**
Juice (g)	16,250	58,1637	15,000	67,0820	0,962	0,335**
Vegetables (% recommended daily intake)	107,345	62,6457	160,091	84,3996	0,051	0,740*
Vegetables (g)	375,706	219,2604	560,321	295,3988	0,051	0,740*
Trans-unsaturated fatty acids (g)	1,799	1,3487	1,562	1,0549	0,575	0,943*
Carbohydrates (% caloric intake)	42,265	7,5198	41,955	10,6753	0,912	0,712*
Carbohydrates (g)	215,439	51,5412	212,542	62,2162	0,889	0,903**
Total fats (% caloric intake)	38,224	6,8268	36,799	9,1744	0,556	0,530*
Total fats (g)	86,576	19,6686	84,755	30,9805	0,829	0,561*
Vit. B1 (% recommended daily intake)	196,089	95,7253	193,617	93,9826	0,951	0,726*
Vit. B1 (mg)	1,510	0,7364	1,492	0,7229	0,951	0,726*
Vit. B12 (% recommended daily intake)	108,933	63,7448	170,230	202,4766	0,215	0,710**
Vit. B12 (mg)	4,357	2,5501	6,809	8,0994	0,215	0,710**
Vit. B2 (% recommended daily intake)	206,257	70,9967	163,671	58,6162	0,171	1,000*
Vit. B2 (mg)	1,877	0,6459	1,488	0,5329	0,171	1,000*
Vit. B6 (% recommended daily intake)	187,512	183,1139	196,478	77,6274	0,892	0,308**
Vit. B6 (mg)	1,994	1,9153	1,988	0,8135	0,994	0,291**
Vit. C (% recommended daily intake)	142,199	89,4487	177,704	86,2466	0,352	0,836*
Vit. C (mg)	85,293	42,4830	99,474	43,7323	0,408	0,735*
Vit. D (% recommended daily intake)	13,857	15,3065	42,549	52,0123	0,050	0,416*
Vit. D (mcg)	2,079	2,2959	6,380	7,8023	0,050	0,416*
Vit. E (% recommended daily intake)	125,417	74,3475	159,623	99,9404	0,392	0,651**
Vit. E (mg)	16,304	9,6656	20,752	12,9913	0,392	0,651**
Fruits (% recommended daily intake)	134,779	75,1886	141,259	97,2922	0,827	0,281**
Fruits (g)	269,557	150,3767	282,518	194,5844	0,827	0,281**
Saturated fatty acids (% caloric intake)	16,160	6,0581	12,030	4,2199	0,058	0,299**
Saturated fatty acids (g)	36,627	13,9657	27,927	11,6296	0,058	0,284**
Zn (% recommended daily intake)	95,898	29,5376	105,192	42,8322	0,491	0,095**
Zn (mg)	15,344	4,7260	16,831	6,8536	0,491	0,095**
Grains (% recommended daily intake)	99,972	49,4513	104,099	83,6490	0,872	0,519**
Grains (g)	231,937	114,7272	241,512	194,0641	0,872	0,519**

DII= dietary inflammatory index. [a] The cut-off for late responders was 9 or more months to reach complete response.
* Huynh-Feldt **Greenhouse-Geisser

Supplementary Table S8: Univariate logistic regression analysis on dietary components previously shown to be associated with significant difference between metastatic melanoma patients with early and late response to immunotherapy.

Dietary component	Univariate odds ratio	95% Confidence interval	p-value
Alcohol (g/day)	1,0061	1,0008 to 1,0116	0,0248
Anthocyanin (mg/day)	1,0019	0,9998 to 1,0040	0,0742
Flavones (mg/day)	0,9996	0,9839 to 1,0157	0,9655
Potatoes (g/day)	1,0043	1,0003 to 1,0082	0,0329
Polyunsaturated fatty acids (% energy intake)	1,2684	1,0628 to 1,5139	0,0084
Proteins (% recommended protein (g) use per body weight (kg))	0,9714	0,9535 to 0,9896	0,0022
Sweets (g/day)	0,9747	0,9563 to 0,9934	0,0084

All vegetable (g/day)	1,0102	1,0022 to 1,0184	0,0130
Vitamin D (mcg/day)	1,0332	1,0033 to 1,0641	0,0293
Saturated fatty acids (% energy intake)	0,8481	0,7453 to 0,9651	0,0125

Supplementary Table S9: Correlation coefficients (r) between selected dietary components and absolute abundance of selected bacteria on phylum, family and species levels.

	Recomm. protein intake (% day) (p)	SFA (g/day) (p)	PUFA (% en. intake) (p)	Fibres (g/day) (p)	Vitamin D (mcg/day) (p)	Sodium (g/day) (p)	All vegetables (g/day) (p)	Sweets (g/day) (p)	Flavones (mcg/day) (p)	Antho-cyanin (mcg/day) (p)	Alcohol (g/day)	Potato (g/dy)
PHYLUM LEVEL												
Actinobacteria	-0.32 (0.24)	0.17 (0.53)	-0.06 (0.83)	0.20 (0.46)	0.09 (0.72)	0.46 (0.08)	0.50 (0.06)	-0.23 (0.40)	0.05 (0.86)	0.21 (0.44)	0.02 (0.94)	0.34 (0.22)
Firmicutes	-0.19 (0.49)	0.10 (0.71)	0.29 (0.29)	0.05 (0.85)	0.46 (0.09)	0.07 (0.79)	-0.04 (0.89)	<-0.01 (0.98)	0.15 (0.59)	0.56 (0.03)	0.27 (0.33)	0.68 (<0.01)
Bacteroidetes	-0.06 (0.83)	0.27 (0.32)	0.22 (0.43)	0.11 (0.71)	0.37 (0.18)	0.14 (0.63)	-0.41 (0.13)	0.36 (0.18)	-0.25 (0.36)	-0.32 (0.25)	-0.39 (0.15)	-0.03 (0.90)
Proteobacteria	0.12 (0.66)	0.41 (0.13)	0.01 (0.97)	<0.01 (0.97)	-0.09 (0.75)	0.14 (0.61)	-0.05 (0.87)	0.43 (0.10)	-0.17 (0.54)	-0.38 (0.16)	-0.21 (0.45)	<-0.01 (0.98)
FAMILY LEVEL												
Prevotellaceae	0.33 (0.23)	0.39 (0.14)	-0.29 (0.31)	0.04 (0.88)	-0.15 (0.60)	0.38 (0.16)	-0.24 (0.38)	0.47 (0.08)	-0.15 (0.59)	-0.37 (0.17)	-0.16 (0.57)	-0.23 (0.40)
Bacteroidaceae	-0.30 (0.28)	-0.11 (0.69)	0.53 (0.04)	0.07 (0.79)	0.69 (<0.01)	-0.16 (0.58)	-0.38 (0.16)	0.02 (0.94)	-0.20 (0.46)	-0.02 (0.93)	-0.36 (0.19)	0.05 (0.85)
Bifidobacteriaceae	-0.35 (0.19)	0.24 (0.39)	<0.01 (0.99)	0.27 (0.33)	0.17 (0.54)	0.40 (0.14)	0.38 (0.15)	-0.21 (0.45)	-0.14 (0.61)	0.22 (0.42)	-0.23 (0.41)	0.36 (0.18)
Clostridiales	-0.36 (0.19)	0.07 (0.79)	0.35 (0.19)	-0.23 (0.40)	0.23 (0.41)	-0.04 (0.87)	-0.27 (0.33)	0.06 (0.83)	-0.11 (0.70)	0.19 (0.49)	0.03 (0.90)	0.51 (0.05)
Lactobacillaceae	-0.08 (0.77)	-0.08 (0.76)	-0.14 (0.62)	-0.19 (0.49)	0.11 (0.68)	0.05 (0.85)	0.24 (0.39)	-0.03 (0.92)	0.54 (0.04)	-0.05 (0.87)	0.64 (0.01)	-0.09 (0.75)
Akkermansiaceae	-0.14 (0.62)	-0.07 (0.79)	-0.21 (0.45)	0.14 (0.61)	-0.18 (0.51)	0.35 (0.19)	0.20 (0.47)	-0.37 (0.17)	0.32 (0.24)	-0.17 (0.54)	0.18 (0.52)	-0.33 (0.23)
Ruminococcaceae	-0.35 (0.19)	-0.25 (0.37)	0.08 (0.77)	-0.13 (0.64)	0.11 (0.69)	0.16 (0.55)	0.31 (0.26)	-0.22 (0.43)	0.56 (0.03)	0.32 (0.24)	0.79 (<0.01)	0.46 (0.09)
Lachnospiraceae	-0.21 (0.46)	0.07 (0.79)	0.34 (0.21)	0.17 (0.54)	0.51 (0.05)	0.09 (0.74)	<-0.01 (0.99)	-0.15 (0.59)	0.13 (0.63)	0.42 (0.12)	<0.01 (0.98)	0.58 (0.02)
Coriobacteriaceae	0.48 (0.07)	-0.07 (0.81)	-0.18 (0.50)	-0.22 (0.44)	-0.22 (0.42)	-0.24 (0.38)	0.12 (0.67)	0.24 (0.38)	0.04 (0.88)	-0.05 (0.86)	0.45 (0.09)	-0.18 (0.52)
SPECIES LEVEL												
Coprococcus comes	-0.33 (0.22)	-0.12 (0.67)	0.17 (0.55)	-0.26 (0.34)	0.47 (0.07)	0.11 (0.69)	0.13 (0.64)	-0.08 (0.78)	0.27 (0.32)	0.31 (0.25)	0.29 (0.29)	0.45 (0.09)
Bifidobacterium pseudocatenulatum	-0.48 (0.07)	-0.16 (0.58)	0.51 (0.05)	0.11 (0.68)	0.74 (<0.01)	0.32 (0.24)	-0.03 (0.91)	-0.39 (0.15)	0.18 (0.52)	0.20 (0.47)	<-0.01 (0.98)	0.19 (0.50)
Barnesiella intestinihominis	0.02 (0.93)	0.21 (0.46)	0.03 (0.92)	0.19 (0.48)	-0.18 (0.51)	0.07 (0.80)	-0.18 (0.52)	0.23 (0.40)	-0.17 (0.55)	-0.39 (0.14)	-0.27 (0.34)	-0.22 (0.43)
Sutterella wadsworthensis	0.39 (0.15)	0.01 (0.96)	-0.19 (0.47)	-0.06 (0.82)	-0.18 (0.52)	-0.48 (0.06)	-0.23 (0.40)	0.24 (0.39)	-0.17 (0.55)	-0.24 (0.39)	0.14 (0.61)	-0.17 (0.55)
Bacteroides finegoldii	0.19 (0.49)	0.42 (0.12)	-0.26 (0.35)	-0.17 (0.55)	-0.17 (0.55)	-0.27 (0.33)	-0.34 (0.21)	0.49 (0.06)	-0.21 (0.44)	-0.51 (0.05)	-0.31 (0.25)	-0.39 (0.15)
Dark grey shades further indicate p<0.05, and light gray p=0.05-0.10. SFA = saturated fatty acids.PUFA = unsaturated fatty acids.												

Supplementary Table S10: Correlation coefficients (r) between selected dietary components and relative abundance of selected bacteria on phylum, family and species levels.

	Recomm. protein intake (% day) (p)	SFA (g/day) (p)	PUFA (% en. intake) (p)	Fibres (g/day) (p)	Vitamin D (mcg/day) (p)	Sodium (g/day) (p)	All vegetables (g/day) (p)	Sweets (g/day) (p)	Flavones (mcg/day) (p)	Antho-cyanin (mcg/day) (p)	Alcohol (g/day)	Potato (g/dy)
PHYLUM LEVEL												
Actinobacteria	-0.32 (0.23)	0.02 (0.94)	-0.21 (0.45)	0.13 (0.63)	-0.12 (0.67)	0.39 (0.15)	0.67 (<0.01)	-0.35 (0.19)	0.11 (0.69)	0.14 (0.63)	0.09 (0.72)	0.11 (0.69)
Firmicutes	-0.03 (0.90)	-0.28 (0.31)	-0.01 (0.96)	-0.07 (0.81)	-0.06 (0.82)	-0.11 (0.68)	0.36 (0.18)	-0.33 (0.23)	0.36 (0.19)	0.52 (0.04)	0.52 (0.046)	0.29 (0.29)
Bacteroidetes	0.07 (0.80)	0.25 (0.37)	0.07 (0.81)	0.05 (0.86)	0.11 (0.71)	0.04 (0.89)	-0.46 (0.08)	0.35 (0.20)	-0.35 (0.19)	-0.46 (0.08)	-0.51 (0.05)	-0.26 (0.35)
Proteobacteria	0.10 (0.71)	0.32 (0.25)	-0.06 (0.82)	-0.05 (0.86)	-0.20 (0.47)	0.07 (0.79)	<0.01 (0.99)	0.32 (0.25)	-0.16 (0.57)	-0.43 (0.11)	-0.18 (0.52)	-0.09 (0.73)
FAMILY LEVEL												
Prevotellaceae	0.33 (0.23)	0.39 (0.14)	-0.28 (0.31)	0.04 (0.88)	-0.15 (0.60)	0.38 (0.16)	-0.24 (0.38)	0.47 (0.08)	-0.15 (0.59)	-0.37 (0.17)	-0.13 (0.66)	-0.24 (0.44)
Bacteroidaceae	-0.30 (0.28)	-0.11 (0.69)	0.53 (0.04)	0.07 (0.79)	0.69 (<0.01)	-0.16 (0.58)	-0.38 (0.16)	0.02 (0.93)	-0.20 (0.46)	-0.02 (0.93)	-0.47 (0.08)	-0.16 (0.56)
Bifidobacteriaceae	-0.35 (0.19)	0.24 (0.39)	<0.01 (0.99)	0.27 (0.33)	0.17 (0.54)	0.40 (0.14)	0.39 (0.15)	-0.21 (0.45)	-0.14 (0.61)	0.22 (0.41)	-0.23 (0.41)	0.24 (0.38)
Clostridiales	-0.36 (0.19)	0.07 (0.79)	0.35 (0.19)	-0.23 (0.40)	0.23 (0.41)	-0.04 (0.88)	-0.27 (0.33)	0.06 (0.83)	-0.11 (0.70)	0.19 (0.49)	-0.02 (0.95)	0.24 (0.39)
Lactobacillaceae	-0.08 (0.77)	-0.08 (0.76)	-0.14 (0.61)	-0.19 (0.49)	0.12 (0.68)	0.05 (0.85)	0.24 (0.39)	-0.03 (0.92)	0.53 (0.04)	-0.05 (0.87)	0.67 (<0.01)	-0.21 (0.48)
Akkermansiaceae	-0.14 (0.62)	-0.07 (0.79)	-0.21 (0.45)	0.14 (0.62)	-0.18 (0.51)	0.35 (0.19)	0.20 (0.47)	-0.37 (0.17)	0.32 (0.24)	-0.17 (0.54)	0.19 (0.48)	-0.37 (0.17)

Ruminococcaceae	-0.35 (0.19)	-0.25 (0.37)	0.08 (0.78)	-0.13 (0.64)	0.11 (0.69)	0.17 (0.55)	0.31 (0.26)	-0.22 (0.42)	0.56 (0.03)	0.32 (0.24)	0.70 (<0.01)	-0.04 (0.88)
Lachnospiraceae	-0.21 (0.46)	0.07 (0.79)	0.34 (0.21)	0.17 (0.54)	0.51 (0.05)	0.09 (0.74)	<-0.01 (0.99)	-0.15 (0.59)	0.13 (0.63)	0.42 (0.12)	0.09 (0.74)	0.32 (0.24)
Coriobacteriaceae	0.48 (0.07)	-0.07 (0.81)	-0.19 (0.50)	-0.22 (0.44)	-0.22 (0.42)	-0.24 (0.38)	0.12 (0.67)	0.24 (0.38)	0.04 (0.88)	-0.05 (0.86)	0.46 (0.08)	-0.35 (0.20)
SPECIES LEVEL												
Coprococcus comes	-0.24 (0.39)	-0.26 (0.34)	<0.01 (0.99)	-0.36 (0.18)	0.19 (0.48)	0.04 (0.89)	0.35 (0.19)	-0.15 (0.59)	0.34 (0.21)	0.31 (0.27)	0.40 (0.14)	0.23 (0.41)
Bifidobacterium pseudocatenulatum	-0.51 (0.05)	-0.14 (0.63)	0.36 (0.18)	0.11 (0.69)	0.53 (0.04)	0.46 (0.09)	0.10 (0.71)	-0.47 (0.08)	0.29 (0.29)	0.17 (0.54)	0.12 (0.67)	0.13 (0.63)
Barnesiella intestinihominis	<0.01 (0.99)	0.17 (0.55)	-0.01 (0.96)	0.19 (0.48)	-0.19 (0.49)	0.04 (0.88)	-0.21 (0.45)	0.14 (0.62)	-0.13 (0.64)	-0.41 (0.13)	-0.27 (0.32)	-0.25 (0.36)
Sutterella wadsworthensis	0.38 (0.16)	-0.04 (0.88)	-0.17 (0.55)	-0.08 (0.78)	-0.17 (0.53)	-0.47 (0.08)	-0.22 (0.42)	0.19 (0.49)	-0.15 (0.58)	-0.28 (0.31)	0.16 (0.57)	-0.19 (0.48)
Bacteroides finegoldii	0.19 (0.51)	0.37 (0.17)	-0.29 (0.29)	-0.16 (0.58)	-0.17 (0.53)	-0.28 (0.32)	-0.37 (0.17)	0.41 (0.13)	-0.18 (0.52)	-0.51 (0.05)	-0.33 (0.23)	-0.42 (0.12)
Dark grey shades further indicate p<0.05, and light gray p=0.05-0.10. SFA = saturated fatty acids. PUFA = unsaturated fatty acids.												
